# Automatic Detection and Classification of Modic Changes in MRI Images Using Deep Learning: Intelligent Assisted Diagnosis System

**DOI:** 10.1111/os.13894

**Published:** 2023-11-07

**Authors:** Gang Liu, Lei Wang, Sheng‐nan You, Zhi Wang, Shan Zhu, Chao Chen, Xin‐long Ma, Lei Yang, Shuai Zhang, Qiang Yang

**Affiliations:** ^1^ Clinical School/College of Orthopaedics Tianjin Medical University Tianjin China; ^2^ Department of Spine Surgery, Tianjin Hospital Tianjin University Tianjin China; ^3^ State Key Laboratory of Reliability and Intelligence of Electrical Equipment, School of Health Sciences & Biomedical Engineering Hebei University of Technology Tianjin China

**Keywords:** End‐plate osteochondritis, Magnetic resonance imaging, Modic changes, ResNet18, SSD

## Abstract

**Objective:**

Modic changes (MCs) are the most prevalent classification system for describing intravertebral MRI signal intensity changes. However, interpreting these intricate MRI images is a complex and time‐consuming process. This study investigates the performance of single shot multibox detector (SSD) and ResNet18 network‐based automatic detection and classification of MCs. Additionally, it compares the inter‐observer agreement and observer‐classifier agreement in MCs diagnosis to validate the feasibility of deep learning network‐assisted detection of classified MCs.

**Method:**

A retrospective analysis of 140 patients with MCs who underwent MRI diagnosis and met the inclusion and exclusion criteria in Tianjin Hospital from June 2020 to June 2021 was used as the internal dataset. This group consisted of 55 males and 85 females, aged 25 to 89 years, with a mean age of (59.0 ± 13.7) years. An external test dataset of 28 patients, who met the same criteria and were assessed using different MRI equipment at Tianjin Hospital, was also gathered, including 11 males and 17 females, aged 31 to 84 years, with a mean age of 62.7 ± 10.9 years. After Physician 1 (with 15 years of experience) annotated all MRI images, the internal dataset was imported into the deep learning model for training. The model comprises an SSD network for lesion localization and a ResNet18 network for lesion classification. Performance metrics, including accuracy, recall, precision, F1 score, confusion matrix, and inter‐observer agreement parameter Kappa value, were used to evaluate the model's performance on the internal and external datasets. Physician 2 (with 1 year of experience) re‐labeled the internal and external test datasets to compare the inter‐observer agreement and observer‐classifier agreement.

**Results:**

In the internal dataset, when models were utilized for the detection and classification of MCs, the accuracy, recall, precision and F1 score reached 86.25%, 87.77%, 84.92% and 85.60%, respectively. The Kappa value of the inter‐observer agreement was 0.768 (95% CI: 0.656, 0.847),while observer‐classifier agreement was 0.717 (95% CI: 0.589, 0.809).In the external test dataset, the model's the accuracy, recall, precision and F1 scores for diagnosing MCs reached 75%, 77.08%, 77.80% and 74.97%, respectively. The inter‐observer agreement was 0.681 (95% CI: 0.512, 0.677), and observer‐classifier agreement was 0.519 (95% CI: 0.290, 0.690).

**Conclusion:**

The model demonstrated strong performance in detecting and classifying MCs, achieving high agreement with physicians in MCs diagnosis. These results suggest that deep learning models have the potential to facilitate the application of intelligent assisted diagnosis techniques in the field of spine research.

## Graphical abstract

We evaluated the performance of SSD and ResNet18 network‐based automatic detection and classification of MCs. Additionally, it compares the inter‐observer agreement in MCs diagnosis and the agreement between observers and classifiers to validate the feasibility of deep learning network‐assisted detection of classified MCs.

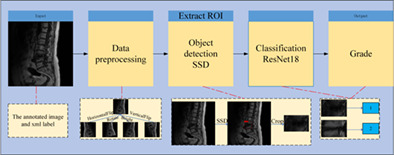

## Introduction

Low back pain (LBP) is a prevalent musculoskeletal problem among working‐age individuals^1^ and is associated with functional impairment, decreased quality of life, and increased health care costs.^2^, ^3^ In China, the number of people suffering from LBP is growing annually and affecting younger populations, indicating a significant public health concern.^4^ Research has demonstrated that degeneration of intervertebral discs and vertebral endplates can contribute to LBP.^5^, ^6^, ^7^, ^8^ Magnetic resonance imaging (MRI) of the lumbar spine is a useful tool for identifying potential sources of LBP, facilitating diagnosis and treatment.^9^, ^10^ Degeneration of the lumbar endplate can result in signal changes on MRI, known as Modic changes (MCs), which are commonly used to classify alterations in the bone marrow of vertebrae adjacent to the endplate.^11^ Endplate inflammation can be clinically categorized into healed, stable, transitional and active stages. This inflammation is commonly observed in the lumbar spine, especially in L4/5 and L5/S1,^12^ and accurate staging of MCs is essential for determining endplate inflammation at various stages.

**FIGURE 1:**
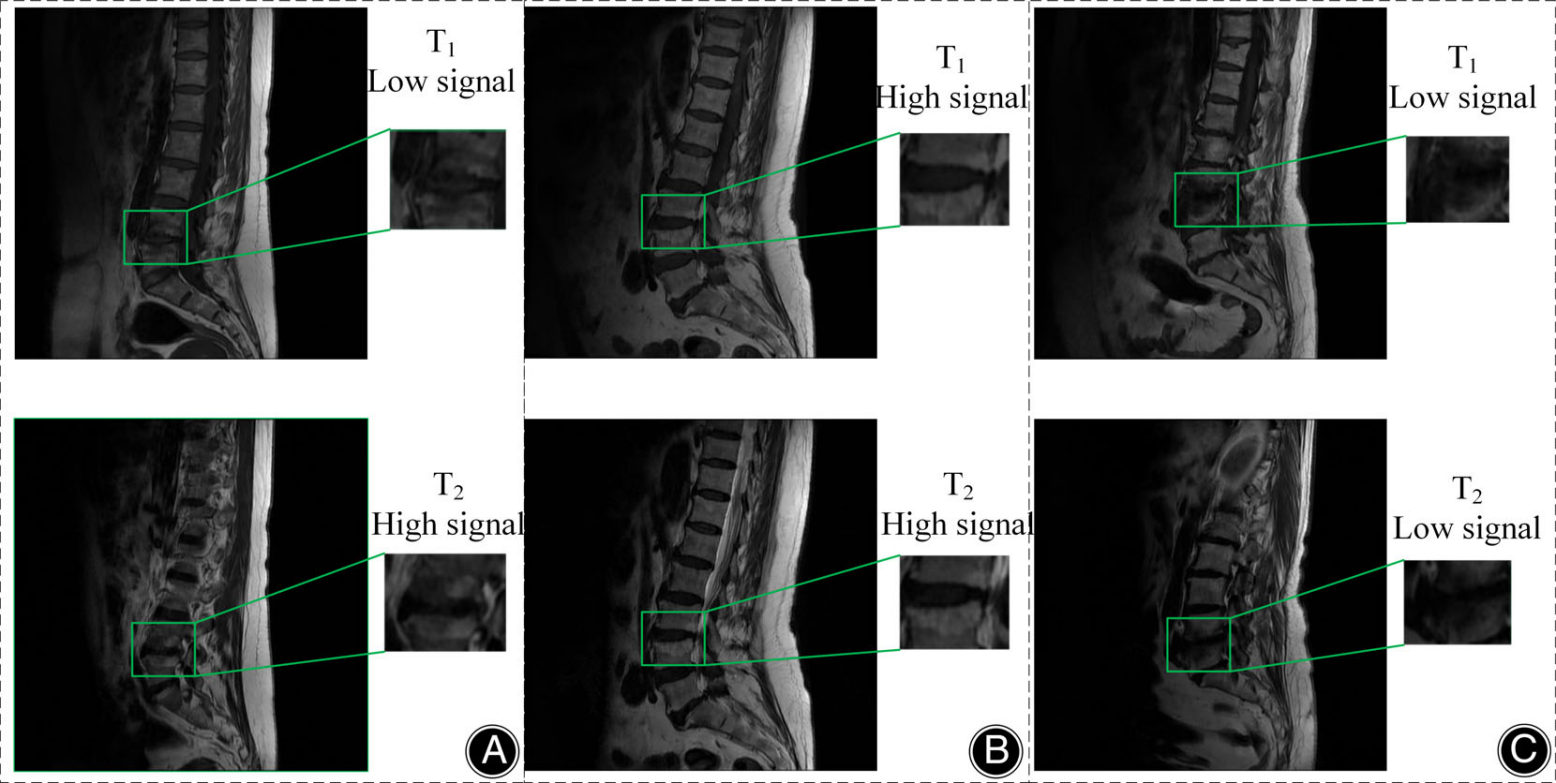
Schematic diagram of the three types of Modic changes. (A) Modic type 1.a low signal in sagittal T1WI and a high signal in T2WI (B) Modic type 2.a high signal in sagittal T1WI and equal/high signal in T2WI (C) Modic type 3.a low signal in sagittal T1WI and T2WI.

Based on the T1‐weighted and T2‐weighted MRI presentations, Modic *et al*. classified MCs into three types: type 1, type 2, and type 3. As illustrated in Figure 1A, the histology of type 1 MCs reveals cartilage lamellar fissure formation and subchondral fibrous tissue production.^11^ MRI imaging shows a low signal in sagittal T1WI and a high signal in T2WI, with endplate inflammation in the active phase. Figure 1B demonstrates that the histology of type 2 MCs exhibits a process of fatty infiltration within the adjacent vertebral body.^11^ MRI imaging presents a high signal in sagittal T1WI and equal/high signal in T2WI, with inflammation in a stable phase. As shown in Figure 1C, the histology of type 3 MCs shows fibrosis and calcification in the adjacent vertebral body,^11^ MRI images reveal a low signal in sagittal T1WI and T2WI, with the lesion in the healing phase. The correlation between MCs and LBP remains unclear and controversial.^13^, ^14^ However, some studies have identified a positive correlation between MCs and LBP,^15^, ^16^ particularly type 1 MCs.^17^

The high prevalence of endplate inflammation has resulted in a large number of MRI images, exceeding the number of physicians available to interpret them. Interpreting these MRI images is repetitive and time‐consuming task, even for the most specialized radiologists. As the population ages, the incidence of endplate inflammation is gradually increasing. To address these challenges, automated methods for diagnosing and classifying endplate inflammation need to be explored. Recent advancements in artificial intelligence have enabled physicians to employ computer algorithms to tackle these laborious tasks, significantly reducing their workload.

Deep learning models, especially convolutional neural network (CNN) models, have been effectively employed for target detection and classification across various disciplines, including radiology,^18^, ^19^ pathology,^20^ dermatology,^21^ and ophthalmology.^22^ Significant progress has been made in researching computer‐aided diagnosis of endplate diseases using CNNs. In 2017, Jamaludin *et al*.^23^ developed SpineNet, which can detect endplate defects and bone marrow alterations from MRI with a high level of accuracy. However, the study only investigated the presence of bone marrow signal changes without specifying MRI signal changes. In 2021, Gao *et al*.^24^ examined consistency differences in physicians' diagnostic MCs staging with and without deep learning model assistance, revealing increased inter‐rater agreement scores when using a deep learning model. In 2022, Windsor *et al*.^25^ proposed the SpineNetv2 network, offering faster vertebral phase detection than the SpineNet network but only exploring bone marrow signal changes without specific MRI signal change determination. In 2023, Wang *et al*.^26^ compared the performance of using only the Yolov5 network *versus* using both Yolov5 and Resnet34 for detecting classified MCs, demonstrating superior performance with the two‐network approach, However, the study did not investigate the consistency between the model and physician diagnosis. This study builds upon Wang *et al*.'s^26^ research by exploring interobserver agreement and the concordance between the model and physician diagnosis.

The aforementioned studies have made varying degrees of progress, and this study examines the performance of single shot multibox detector (SSD) and ResNet18 networks for automatic detection and classification of MCs. The study content is divided into the following three parts: (i) this study investigates the use of SSD network for lesion area localization and ResNet18 for lesion area classification. Notably, the performance of the SSD network in detecting spinal disorders has not been explored; (ii) the study compares diagnostic agreement between physicians and between physicians and models; and (iii) external validation was conducted using datasets obtained from different types of MRI devices to assess the performance of deep learning models in assisting with MCs diagnosis.

## Materials and Methods

### Data Set Preparation

A retrospective analysis of 200 patients who underwent lumbar spine MRI diagnosis at Tianjin Hospital from June 2020 to June 2021 was used as an internal data set, which is used to train the model. Inclusion criteria were: (i) patients aged 19 years or older; and (ii) presence of acute to chronic LBP, radiculopathy, and other lumbar spine symptoms, including numbness, tingling, weakness and abnormal sensation. Exclusion criteria were: (i) vertebral fracture; (ii) post‐lumbar internal fixation; (iii) primary tumor; (iv) metastatic spinal disease; and (v) infection. Sixty patients were excluded based on the exclusion criteria, leaving 140 patients in the study, with 55 males and 85 females, aged 25–89 years, with a mean age of 59.0 ± 13.7 years.

Additionally, An external dataset was selected to verify the generalizability of the model a retrospective analysis of 40 patients who underwent lumbar MRI diagnosis at Tianjin Hospital from June 2020 to June 2021 was conducted, with 12 patients excluded according to the exclusion criteria. The remaining 28 patients were included in the external test dataset, as shown in Table 1. Different types of MRI equipment were used for MRI data from external and internal datasets.

**TABLE 1:** Patients' demographic

Characteristics	Internal dataset (*n* = 140)	External test dataset (*n* = 28)
Age (years)	59.0 ± 13.7	62.7 ± 10.9
Men (cases)	55	11
Women (cases)	85	17

Internal datasets were acquired using a 3.0 T MRI scanner (Ingenia CX, Philips Healthcare, Best, the Netherlands). The T1WI image acquisition parameters for the internal dataset were as follows, repetition time (ms) 583 ms. echo time (ms) 10 ms. field of view 300 × 244 mm^2^, slice thickness(mm) 5 mm. and bandwidth 289 kHz. The T2WI image acquisition parameters for internal dataset were as follows, repetition time(ms): 1069 ms. echo time (ms): 80 ms. field of view (mm^2^): 300 × 244 mm^2^. slice thickness (mm) 5 mm, bandwidth (kHz) 294 kHz. MRI images were stored as DICOM files. External datasets were acquired using a 3.0 T MRI scanner (United Imaging, Shanghai, China), with details provided in Table 2. The study was approved by the Ethics Committee of Tianjin Hospital (2023 medical ethics108), and informed consent was obtained from all patients.

**TABLE 2:** Summary of the MRI parameter ranges

Brands	Philips		United imaging	
	T_1_‐weighted	T_2_‐weighted	T_1_‐weighted	T_2_‐weighted
Repetition time (ms)	583	1069	1270	2200
Echo time (ms)	10	80	30	50
Field of view (mm^2^)	300 × 224	300 × 224	400 × 336	300 × 224
Slice thickness (mm)	5	5	5	5
Bandwidth (KHz)	289	294	205	262

**FIGURE 2:**
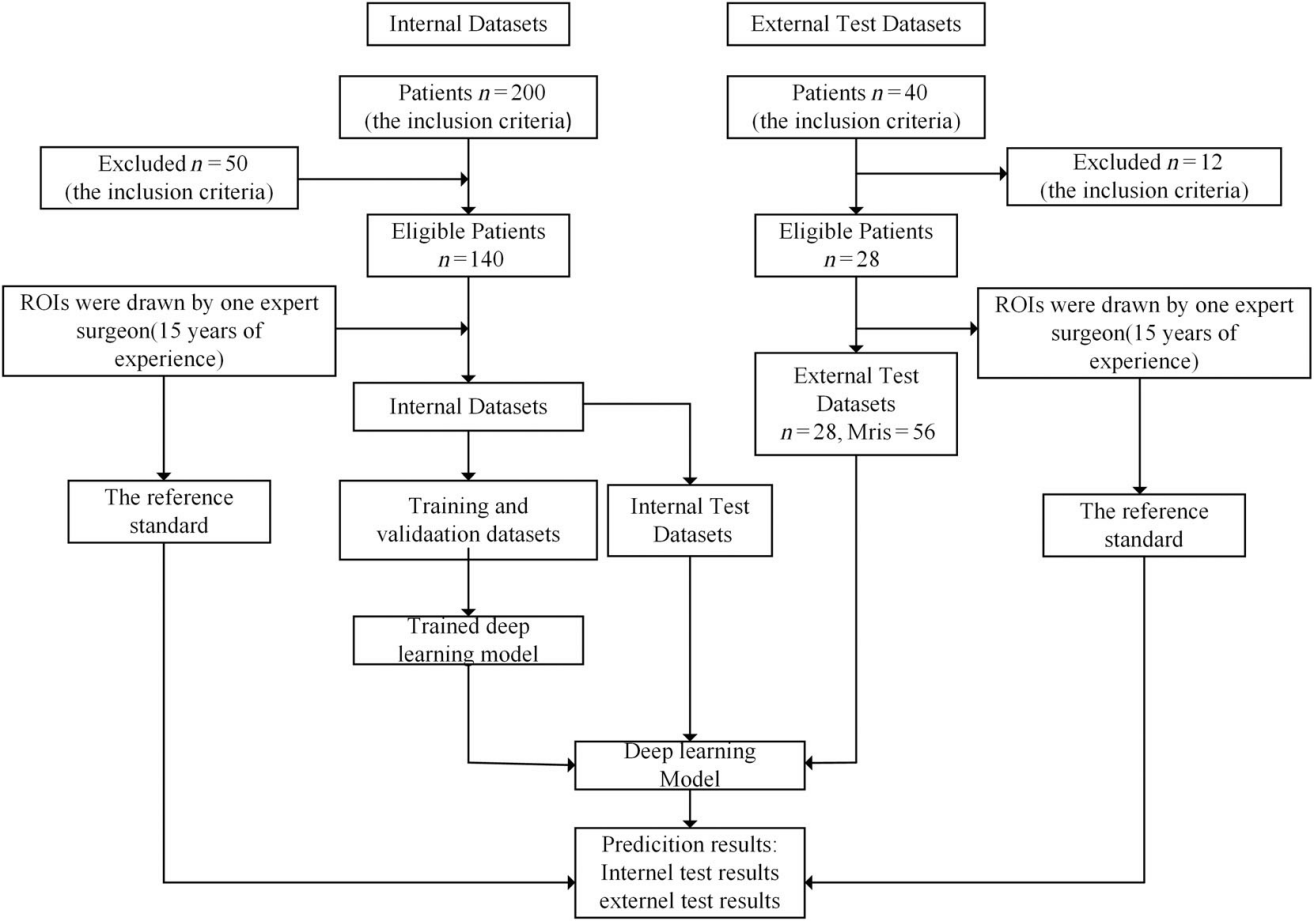
Flowchart showing patients' selection for the datasets and the process of training deep learning model. *n*, number of patients. MRIs, MRI images; ROIs, regions of interest.

The internal dataset used for training the deep learning model comprised 280 images from 140 patients, with median sagittal T1 and T2‐weighted MRI images selected for each patient. T1 and T2 MRI images contained only low signal in 16 patients, only high signal in 60 patients, and low signal in T1 MRI images with high signal in T2 MRI images for 64 patients. Each patient's MRI image contained at least one high or low signal. To train a deep learning model, 280 MRI images are insufficient. Data augmentation is an effective method to reduce overfitting in CNN networks due to limited data. Data enhancement includes methods such as vertical flip, horizontal flip, rotation and brightness change. The augmented images were filtered to exclude those with poor image quality, resulting in a final count of 725 images. The external test dataset contained 56 images from 28 patients, with median sagittal T1‐ and T2‐weighted MRI images selected for each patient. Figure 2 provides a flowchart of the dataset study design.

### Image Annotation

**FIGURE 3:**
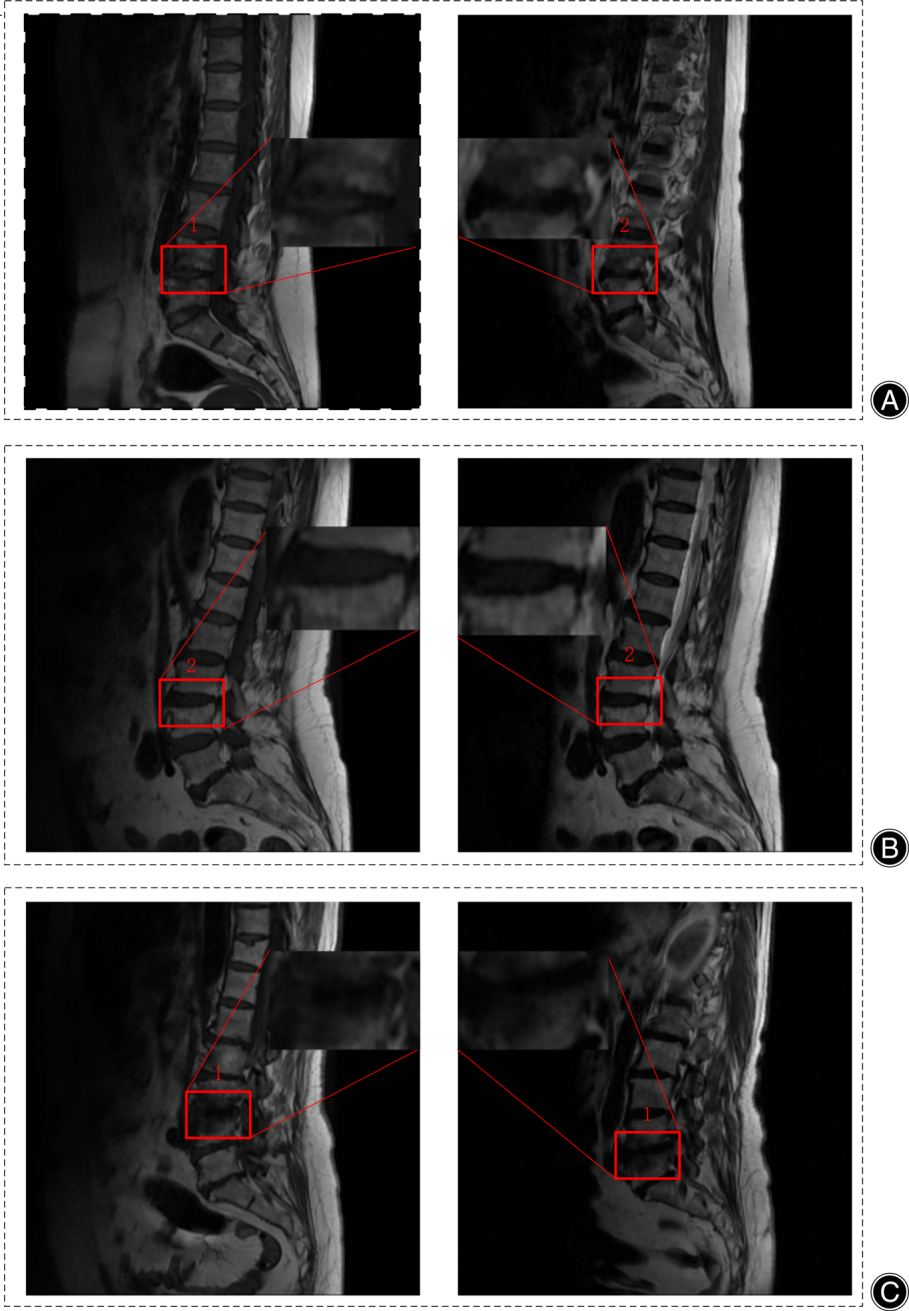
Example of the annotation of the three diseases. (A) Modic type 1. (B) Modic type 2. (C) Modic type 3. Low and high signals are marked with numbers 1 and 2.

Physician 1 (with 15 years of experience) used labeling to label all regions of interest (ROIs) present on the MRI images, with each image containing one or more ROIs labeled by the physician. The physician 1 uses the Modic classification system to label the low and high signals on each MRI sequence with the numbers 1 and 2. Figure 3 illustrates an example of the labeling of high and low signals in three types of diseases. When using Labeling to annotate images, an XML tag file is generated simultaneously, containing the categories and coordinates of the ROIs. Physician 1 independently annotated each sagittal T_1_‐ and T_2_‐weighted MRI image, and the results of these annotations served as a reference standard.

According to MCs typing method,^11^ different types of MCs exhibit variations in high and low signals on MRI. If the deep learning model can accurately identify the high and low signals of the endplate on MRI images, it can accurately classify endplate inflammation.

### Classification Using Deep Learning Model

**FIGURE 4:**
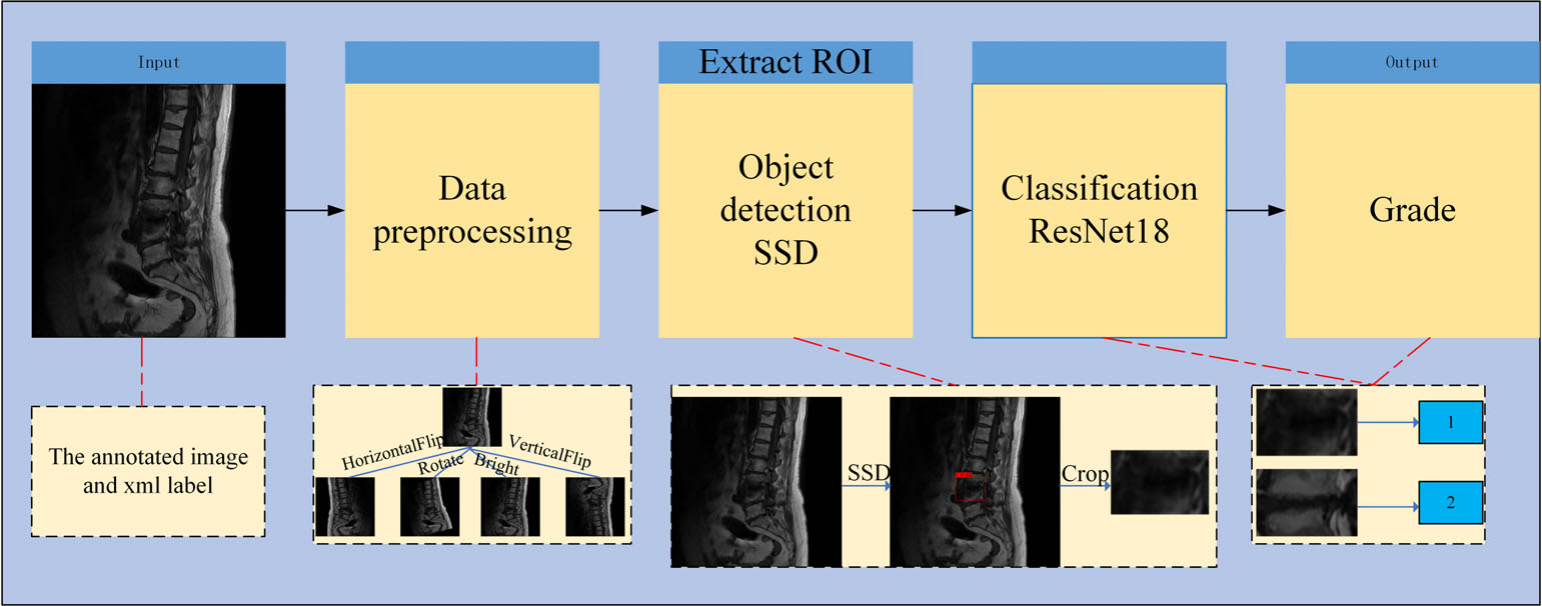
The deep learning model consists of a data preprocessing module, a target detection network, and an image classification network. The MRI graphics undergo preprocessing, which includes vertical flipping, horizontal flipping, rotation by a certain angle, and brightness adjustment. The pre‐processed images are imported into the target detection network SSD, responsible for locating regions of interest in the images, and the localized regions of interest are cropped and imported into the classification network ResNet18 network for classification.

For each label graded by the expert, a classifier is trained to predict high and low signals on MRI. To make the classifier robust, we first localize the lesion regions from MRI images using the target detection network SSD model, and then crop the localized lesion regions. Finally, the classification model RseNet18 predicts the grading. As shown in Figure 4, before the MRI images are annotated and imported into the deep learning model, the MRI graphics undergo preprocessing, which includes vertical flipping, horizontal flipping, rotation by a certain angle, and brightness adjustment. The pre‐processed images are imported into the target detection network SSD, responsible for locating ROI in the images, and the localized ROI are cropped and imported into the classification network ResNet18 network for classification.

In the detection model, we used the SSD^27^ model, which learns the relationship between the image and the bounding box of the lesion region, and is responsible for locating the region of interest in the image. SSD is based on VGGNet^28^ and transforms its FC6 and FC7 layers into convolutional layers, while removing all Dropout and FC8 layers from VGGNet. The SSD algorithm also extends the four convolutional modules, Conv6, Conv7, Conv8, and Conv9, for feature extraction to obtain feature maps with different scales and perceptual fields, enabling detection of objects with varying scales and ensuring high detection accuracy. The SSD network was solely responsible for localizing the lesion area; so the confidence threshold was adjusted to 0.3 to enable the network to predict as many bounding boxes as possible. The physician‐labeled MRI images were randomly divided into a training set (*n* = 580), validation set (*n* = 72) and test set (*n* = 73) in an 8:1:1 ratio and imported into SSD for training. The training set is utilized to build the training parameters, which are continuously updated through several iterations via a gradient descent process. The validation set is used to the current model's accuracy after each epoch is completed. The test set evaluates the model's accuracy based on the validation set's test results. The model is trained for 200 epochs, and every 10 epochs, the model training is saved to obtain the weights. Finally, the training weights for model prediction are selected according to the best accuracy on the validation data, with the batch size set to 32 and a thawing stage of 16.The SGD optimizer was used with a learning rate set to 0.002, a momentum of 0.937, and a weight decay of 0.0005.

For the classification network, we had the bounding box region extracted by an expert and imported into ResNet18 for training. Neural networks are inspired by the human brain and its way of thinking. Humans need to contemplate complex and deep problems, and neural networks simulate solving such problems through deeper networks. However, an increase in network depth may lead to the issue of vanishing gradients. He *et al*. introduced the residual structure in the proposed ResNet network and successfully solved the problem of training accuracy decreases as the number of layers of the network increases, namely, the issue of model degradation.^29^ Commonly used ResNet networks include ResNet18, ResNet34, ResNet50, ResNet101, and ResNet152.

Considering the challenges of image training and the computational capacity of the device in this study, we employed the ResNet18 network. ResNet18 consists of 17 convolutional layers, one maximum pooling layer, one average pooling layer and one fully connected layer. The fully connected layer of ResNet18 is fine‐tuned, while the other layers are loaded with the ResNet18 weight parameters pre‐trained on ImageNet during training. The model is trained for 200 epochs, and every 10 epochs, the weights obtained from the model training are saved. The batch size is set to 8, the SGD optimizer is used, and the learning rate is set to 0.001. As shown in Table 3, the experiments were conducted on a PC with an Intel i5‐8400 2.80 GHz CPU, NVIDIA GTX 1060 and 8 GB RAM, and all algorithms were implemented using PyTorch.

**TABLE 3:** The environment configuration used in the experiment

Environment	Detail
Central processing unit (CPU)	Intel i5‐8400
Operating system	Window 10
Graphic processing unit (GPU)	GTX 1060
PyTorch version	PyTorch 1.8.1
Python	Python 3.9.12
Cuda	11.3
Cudnn	8.0

### Statistical Analysis

For target detection networks, recall (percentage) is used to measure detection performance, as high recall ensures a minimum number of missed ROI bounding boxes. Accuracy, precision, recall and F1 scores were selected as criteria for evaluating the deep model's performance on independent and external test datasets. Accuracy rate indicates the proportion of correct evaluations in all instances; precision rate indicates the proportion of correctly predicted positive samples out of all predicted positive samples; recall represents the percentage of correctly predicted positive samples out of all actual positive samples. The F1 score is a metric that describes a trade‐off between precision and recall, and a larger F1 value indicates better model performance. The confusion matrix summarizes detailed statistics on the classification of deep learning models at the lumbar spine MRI level. The primary outcome measure is the levels of agreement between the DL model and the reference standard for ROI detection and classification. Inter‐observer agreement denotes the degree of diagnosis matching between Physician 1 and Physician 2, while observer–classifier agreement indicates the degree of diagnosis matching between Physician 1 and the trained classifier. Kappa values with 95% confidence intervals (CI) (SPSS, version 25.0, IBM, Armonk, NY, USA) were used to assess the inter‐observer agreement and the clinical reliability of the model. The kappa consistency levels were defined as follows: 0–0.2, poor consistency; 0.21–0.4, fair consistency; 0.41–0.6, moderate consistency; 0.61–0.8, substantial consistency; and 0.81–1, almost‐perfect consistency.

The accuracy, precision, recall and F1 score are calculated as:

(1) Accuracy=TP+TNTP+TN+FP+FN×10000,

(2) Precision=TPTP+FP×10000,

(3) Recall=TPTP+FN×10000,

(4) $$F1=\frac{2P*R}{P+R},$$

where TP, TN, FP and FN represent true positive, true negative, false positive and false negative respectively.

## Results

### Data Volume Statistics Results

**FIGURE 5:**
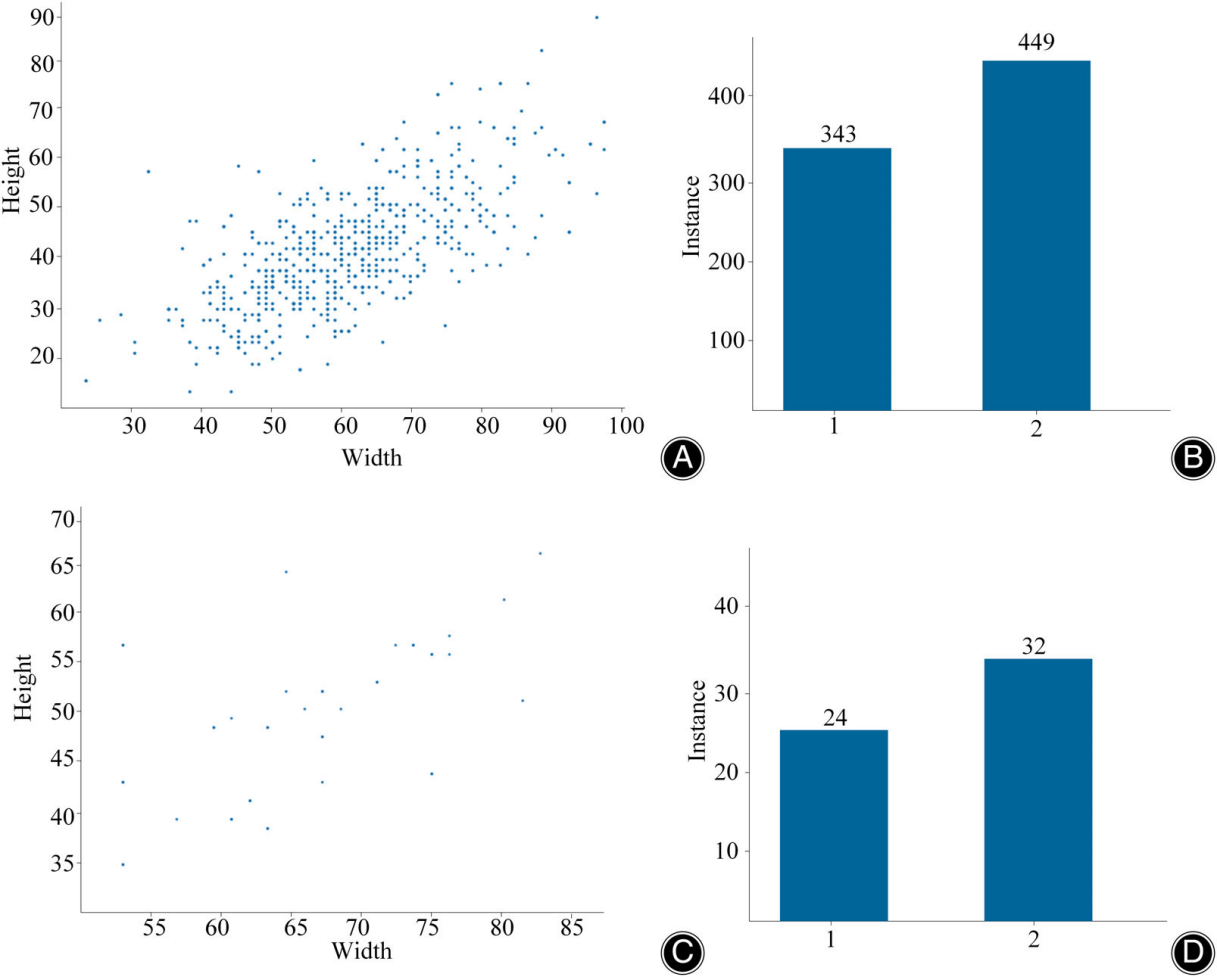
Size distribution of ROIs (A) and distribution of high/low signal (B) on the internal dataset. size distribution of ROIs (C) and distribution of high/low signal (D) on the external dataset. The bounding boxes in the external and internal test sets range roughly 30 × 20 to 100 × 90 on the internal dataset, the number of low and high signals is 343 and 449 on the external dataset, the number of low and high signals is 24 and 32.

The ROI located in the internal and external datasets are extracted and utilized for training of the classification network. However, the size of the bounding boxes may vary due to the different dimensions of the lesion regions, The bounding boxes in the external and internal test sets range roughly 30 × 20 to 100 × 90. In the internal dataset, 343 bounding boxes were labeled 1 and 449 were labeled 2. In the external dataset, 24 bounding boxes are labeled 24 and 32 bounding boxes are labeled 2 As depicted in Figure 5, the statistics of signal class distribution and bounding box feature distribution in the internal and external datasets are presented.

### Consistency Parameters for both Physicians on the Internal Test Set and the External Test Dataset

On both the internal and external test datasets, Physician 2 re‐labeled the data and achieved a high level of consistency with the reference standard. In the internal test dataset, Physician 2 accurately classified 70 of 80 cases, achieving a diagnostic accuracy of 87.5% and a Kappa value of 0.768 (95% CI: 0.656, 0.847) for the concordance parameter with Physician 1. In the external test dataset, Physician 2 accurately classified 47 of 56 cases, with a diagnostic accuracy of 83.93% and an inter‐observer agreement parameter kappa value of 0.681 (95% CI: 0.512, 0.677).

### Performance of Deep Learning Models on the Internal Test Sets

**FIGURE 6:**
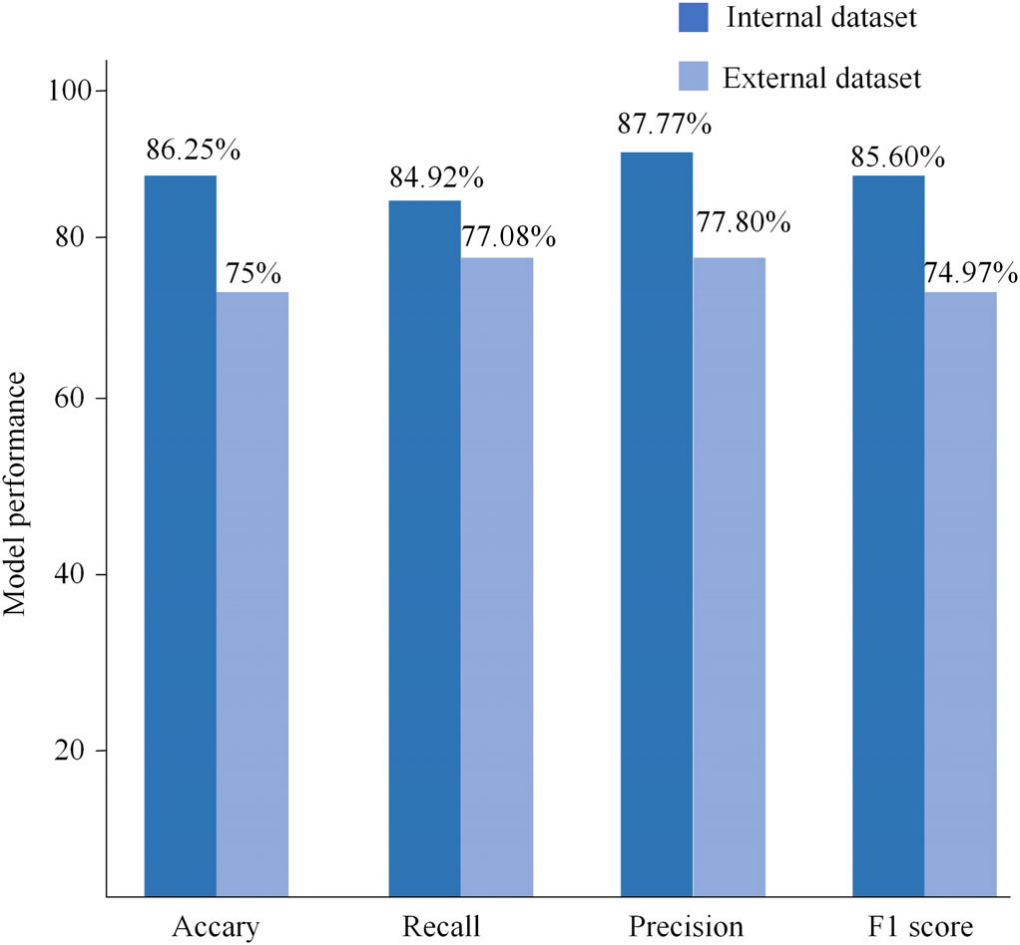
Model performance On the internal and external test sets. On the internal test set, the model classification's accuracy, recall, precision, and F1 scores reached 86.25%, 84.92%, 87.77%, and 85.60%, respectively. The accuracy, recall, precision, and F1 scores of the model classification reached 75%, 77.08%, 77.80%, and 74.97%, respectively.

**FIGURE 7:**
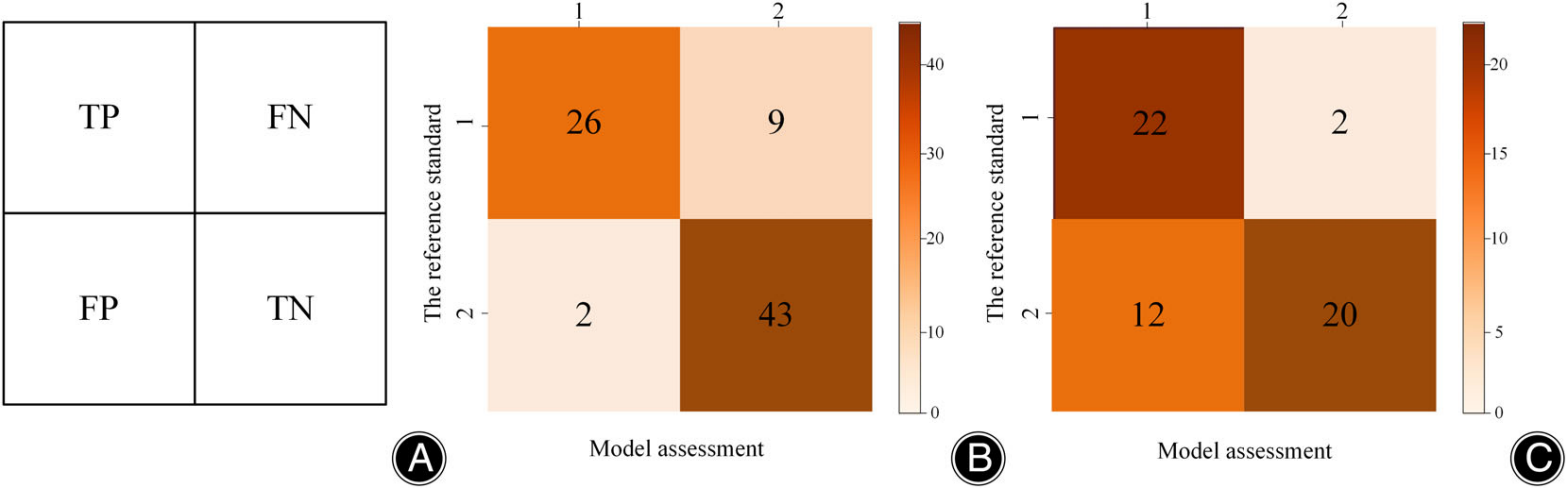
Confusion matrix of the deep‐learning model. (A) Example of confusion matrix. (B) Confusion matrix of the deep‐learning model on the internal dataset. (C) Confusion matrix of the deep‐learning model on the external dataset. TP, TN, FP and FN represent true positive, true negative, false positive and false negative, respectively.

For the target detection network, the SSD network achieved a recall rate of 92.86% for low signals and 88.64% for high signals, with an average recall rate of 90.75%. As depicted in Figure 6, in the internal test set, the model classification's accuracy, recall, precision, and F1 scores reached 86.25%, 84.92%, 87.77%, and 85.60%, respectively. The observer–classifier diagnosis consistency parameter was 0.717 (95% CI: 0.589, 0.809). Figure 7A displays an example of the confusion matrix, where each row represents the true category (reference standard) of the signal in MRI, and the sum of the data in each row represents the total number of that category; each column of the confusion matrix represents the model's predicted category, and the total number of each column signifies the total number of predictions for that category. TP, TN, FP and FN represent true positive, true negative, false positive and false negative, respectively. Figure 7B shows the detailed statistics of the deep learning model's classification on the internal dataset. In the internal dataset, there were 35 low signals and the model correctly predicted 26 of them. There were 45 high signals, of which the model correctly predicted 43.

### Performance of Deep Learning Models on the External Test Sets

For the target detection network, the SSD network achieves a recall of 71.74% for low signals and 73.91% for high signals, with an average recall of 72.82%. As shown in Figure 6, the accuracy, recall, precision, and F1 scores of the model classification reached 75%, 77.08%, 77.80%, and 74.97%, respectively, in the external test set. The observer‐classifier diagnosis consistency parameter yielded a Kappa value of 0.519 (95% CI: 0.290, 0.690). Figure 7C shows the detailed statistics of the deep learning model classification on the external test dataset. In the external dataset, there were 24 low signals, and the model correctly predicted 22 of them. There were 32 high signals, and the model correctly predicted 20 of them.

## Discussions

### Exploring the Performance of SSD Networks

Significant advancements have been made in the development of deep learning algorithms for aiding the diagnosis of spinal disorders. SSD networks have not been explored for diagnosing spinal disorders, and this study examines the performance of SSD and ResNet18 networks for automatic detection and classification of MCs. The deep learning model uses SSD networks for lesion region localization and ResNet18 for lesion region classification. The lesion region localization using SSD network can eliminate edge noise and other irrelevant targets from the original image, solving most noise issues and play a crucial role in the subsequent classification. The research investigates the consistency of inter‐observer diagnosis and observer‐classifier diagnosis, and the results show that the deep learning model performs well in automatic detection and classification of MCs. External validation using datasets obtained from various types of MRI devices was conducted to evaluate the performance of deep learning models in assisting with MCs diagnosis. The results reveal a decrease in both the inter‐observer agreement parameter and the kappa value of the observer–classifier agreement parameter relative to the internal test set, highlighting the necessity for a sufficiently large amount of data and multiple types of MRI images to train deep learning models. To address the issue of limited data, this study uses medical data augmentation for data expansion. CNN models require a large amount of data for training, with the most significant challenge being the scarcity of data for training. Gathering extensive amounts of data, especially medical data, is challenging. Transfer learning^30^ is a research topic in deep learning that focuses on applying pre‐trained model weights to another model to enhance training efficiency and increase accuracy, constructing a high precision model in a short period. Numerous strategies exist for utilizing transfer learning, and fine‐tuning^31^ is one of the more prevalent methods for adapting the model to a specific task. Transfer learning can be used for AI models trained on imagenet natural datasets to fine‐tune the parameters of the AI models using customized medical images.

### Model performance achieves high consistency

Previous research has established the efficacy of deep learning models for classifying diseases displayed in lumbar MRI, and the current study validates the feasibility of using deep learning algorithms to categorize end‐stage inflammation. In 2017, Jamaludin *et al*.^23^ developed SpineNet, which can detect endplate disease from MRI, with an accuracy of 86.7%, 88.3%, 89.7%, and 89.1% for detecting upper and lower bone marrow alterations and upper and lower endplate defects. While the study achieved a high accuracy level, it only explored the presence of bone marrow signal changes and endplate defects, without assessing specific signal changes in MRI. In 2021, Gao *et al*.^24^ analyzed the differences in the consistency of physicians' diagnostic MCs typing with or without deep learning model assistance. They utilized V‐net networks for disc localization, MCs localization on discs, and MCs classification. The results indicated that inter‐rater agreement scores increased with the support of a deep learning model. In 2022, Windsor *et al*.^25^ introduced the SpineNetv2 network, which made many changes to the original network, achieving accuracy rates for detecting upper and lower bone marrow changes and upper and lower endplate defects reached 88.9%, 88.2%, 84.9%, and 89.6%, respectively. However, this model also only examined bone marrow signal changes and endplate defects, without making specific judgments about signal changes. Wang *et al*.^26^ in 2023 explored the performance differences between classifying MCs using only the Yolov5 target detection network and classifying MCs using the target detection network Yolov5 and the classification network resnet34. Their findings revealed the Classification of MCs was superior when using both Yolov5 and Resnet34.The map, recall, precision and F1 scores reached 87.56%, 82.05%, 89.44% and 0.845%, respectively, for Yolov5 detection and classification of MCs, and 88.41%, 88.68% and 0.885%, respectively, for Yolov5 and Resnet34 detection and classification of MCs. Although target detection networks can achieve both localization and classification, using two network models for target classification can resolve most noise issues.

However, most automatic classification models utilizing deep learning algorithms only determine the presence or absence of MCs. Therefore, this study evaluates the specific types of MCs. Based on Wang *et al*.'s research.^26^ The study used two networks to automatically detect and classify end‐plate inflammation, that is, the target detection network was used to localize the lesion area, and the localized lesion area was imported into the classification network for classification, Wang *et al*. used Yolov5 for localization of the lesion area. Unlike the study by Wang *et al*., this study explores the feasibility and performance of SSD networks for spine diagnosis and investigates the consistency of inter‐observer diagnosis and observer‐classifier diagnosis, The use of SSD in the diagnosis of end‐plateitis has not yet been explored. The present study achieved high consistency in quantitative evaluation index accuracy, recall, precision, F1 score, kappa value, relative to previous studies. In the internal dataset, the accuracy, recall, precision and F1 scores reached 86.25%, 87.77%, 84.92% and 0.856%, respectively, when SSD and ResNet18 detected and classified MCs. The consistency parameter Kappa value was 0.768 (95% CI: 0.656,0.847) between Physician 1 and Physician 2, and 0.717 (95% CI: 0.589, 0.809) between Physician 1 and the model. In the external test dataset, the accuracy, recall, precision and F1 score reached 75%, 77.08%, 77.80%, and 74.97%, respectively, with an agreement parameter Kappa value of 0.681 (95% CI: 0.512, 0.677) between Physician 1 and Physician 2 and 0.519 for the model and physician 1 (95% CI: 0.290, 0.690). To assess the performance of deep learning models in assisting MCs diagnosis, external validation was conducted using datasets acquired from various MRI devices. Both the quantitative evaluation metrics and the consistency parameter kappa values decreased in the external test dataset, indicating the importance of training the deep learning model with a sufficiently large dataset and diverse MRI images.

### Strengths and Limitations

The diagnosis of MCs based on SSD and ResNet18 achieved high agreement and good performance compared to previous studies; however this study presents several limitations. First, although we used data augmentation, the data volume remains low. Increasing the sample data size could enhance the performance metrics of MCs’ diagnosis. Future work will incorporate more data, potentially leading to improved deep learning model performance. Second, the model is trained on manually labeled data, the diagnostic performance of the model depends on the reference standard. Only one physician annotated the ROI, potentially introducing greater subjectivity into data annotation. In future research, the data will be annotated by multiple physicians, and consensus will serve as the reference standard. In cases of disagreement, physicians will deliberate and determine the final annotation result, thereby minimizing subjective influences. Third, the datasets in this study originate exclusively from Tianjin Hospital of Tianjin University. Although the study uses image data from different MRI devices at Tianjin Hospital, it lacks datasets from other institutions for external validation to assess the model's generalizability. The decline in deep learning model performance on the external test dataset suggests that the use of large data and diverse MRI images can enhance model stability. Therefore, future work will involve training the deep learning model using MRI images from multiple institutions. Finally, The current study has initiated a series of studies for the adjunctive diagnosis of end‐plateitis, and subsequent work will focus on evaluating the value of the studies in reality.

## Conclusion

In conclusion, we proposed a deep learning network‐based automatic detection and classification of MCs in MRI. The findings demonstrate that using SSD with Resnet18 for the automatic detection and typing of MCs is feasible and highly accurate. Additionally, the model has similar consistency with clinicians in terms of classification of MCs. Ultimately, our findings suggest that the SSD network model can facilitate the diagnosis of lumbar spine MCs, thus highlighting the potential of SSD networks in providing intelligent diagnostic assistance for spinal disorders.

## Author Contributions

Q. Yang, S. Zhang, X‐L. Ma, C. Chen, L. Yang: study concept and design. S. Zhu, Z. Wang: Acquisition of data. S‐N. You, G. Liu, L. Wang: analysis and interpretation. G. Liu, L. Wang: drafting the manuscript.

## Conflict of Interest

This must acknowledge: (i) that all authors listed meet the authorship criteria according to the latest guidelines of the International Committee of Medical Journal Editors; and (ii) that all authors are in agreement with the manuscript.

## ETHICS STATEMENT

This study was approved by the Ethics Review Committee of Tianjin Hospital (IRB number:2023 Medical Ethics Approval No.0108). China.
